# Percutaneous Gastrostomies: Associated Complications in PUSH vs. PULL Techniques over 12 Years in a Referral Centre

**DOI:** 10.3390/jcm13071836

**Published:** 2024-03-22

**Authors:** Ana Piñar-Gutiérrez, Lucía González-Gracia, Rocío Vázquez Gutiérrez, Silvia García-Rey, Andrés Jiménez-Sánchez, Irene González-Navarro, Dolores Tatay-Domínguez, Pilar Garrancho-Domínguez, Pablo J. Remón-Ruiz, Antonio J. Martínez-Ortega, Pilar Serrano-Aguayo, María Dolores Giménez-Andreu, Francisco José García-Fernández, Juan Manuel Bozada-García, Verónica Nacarino-Mejías, Álvaro López-Iglesias, José Luis Pereira-Cunill, Pedro Pablo García-Luna

**Affiliations:** 1 UGC Endocrinología y Nutrición, Hospital Universitario Virgen del Rocio, 41013 Sevilla, Spainlugongra@gmail.com (L.G.-G.); rociov25@hotmail.com (R.V.G.); sylvyes_@hotmail.com (S.G.-R.); irenegonzalez1@gmail.com (I.G.-N.); dolores.tatay.sspa@juntadeandalucia.es (D.T.-D.); pgd0025@gmail.com (P.G.-D.); pjremonruiz@gmail.com (P.J.R.-R.); dr.antmarort@gmail.com (A.J.M.-O.); piagua@gmail.com (P.S.-A.); lolesgian@gmail.com (M.D.G.-A.); garcialunapp@yahoo.es (P.P.G.-L.); 2 Unidad de Aparato Digestivo, Hospital Universitario Virgen del Rocio, 41013 Sevilla, Spain; 3 Unidad de Radiodiagnóstico, Hospital Universitario Virgen del Rocio, 41013 Sevilla, Spain

**Keywords:** gastrostomy, complications, enteral nutrition, peritonitis, interventional radiology, endoscopic techniques

## Abstract

**Objectives**: To compare complications associated with percutaneous gastrostomies performed using PUSH and PULL techniques, whether endoscopic (PEG) or radiological (PRG), in a tertiary-level hospital. **Methods**: This was a prospective observational study. Adult patients who underwent percutaneous PULL or PUSH gastrostomy using PEG or PRG techniques at the Virgen del Rocio University Hospital and subsequently followed up in the Nutrition Unit between 2009–2020 were included. X2 tests or Fisher’s test were used for the comparison of proportions when necessary. Univariate analysis was conducted to study risk factors for PRG-associated complications. **Results**: *n* = 423 (PULL = 181; PUSH = 242). The PULL technique was associated with a higher percentage of total complications (37.6% vs. 23.8%; *p* = 0.005), exudate (18.2% vs. 11.2%; *p* = 0.039), and irritation (3.3% vs. 0%; *p* = 0.006). In the total sample, there were 5 (1.1%) cases of peritonitis, 3 (0.7%) gastrocolic fistulas, and 1 (0.2%) death due to complications associated with gastrostomy. Gender, age, and different indications were not risk factors for a higher number of complications. The most common indications were neurological diseases (35.9%), head and neck cancer (29%), and amyotrophic lateral sclerosis (17.2%). **Conclusions**: The PULL technique was associated with more total complications than the PUSH technique, but both were shown to be safe techniques, as the majority of complications were minor.

## 1. Introduction

In patients with inadequate oral intake who maintain gastrointestinal functionality, enteral nutrition has demonstrated superiority over parenteral nutrition due to its trophic effect on the intestine, lower infectious complications, local and systemic immune system stimulation, and lower cost [1]. In cases where nutritional support requires the use of a tube, gastrostomy is the preferred choice if a duration of more than 4 weeks is expected [2].

Major indications for performing gastrostomy include dysphagia related to neurological conditions (stroke, amyotrophic lateral sclerosis (ALS), cerebral palsy, Parkinson’s disease) or oncological conditions (primarily head and neck cancer and stenosing tumors of the upper digestive tract) and malnutrition related to diseases such as short bowel syndrome, polytrauma, prolonged coma, AIDS, or disorders of upper gastrointestinal motility [2,3].

Currently, there are different techniques for its placement: endoscopic gastrostomy, radiological gastrostomy, and laparoscopic gastrostomy. Although endoscopic gastrostomy is usually the technique of choice, radiological gastrostomy is becoming more and more widespread, and its availability has increased in recent years in hospitals around the world. Currently, surgical placement is reserved for cases where endoscopic and radiological techniques are not feasible, as it is more invasive, requires deeper sedation, and entails higher costs. At present, there is insufficient scientific evidence to establish whether radiological gastrostomy techniques produce a greater number of complications than endoscopic techniques in adult patients [4,5].

Percutaneous endoscopic gastrostomy (PEG) was initially described in 1980 by Gauderer and Ponsky [6]. Since then, various methods for its implementation have been developed. In general, all methods share the common concept of inserting the gastrostomy tube through the abdominal wall at the point closest to the stomach, guided by transillumination with an endoscope. Radiological percutaneous gastrostomy (PRG), first described by Preshaw in 1981 [7], is performed with fluoroscopic guidance.

There are primarily two percutaneous gastrostomy techniques: the PULL technique, also known as traction or Ponsky–Gauderer, and the PUSH technique, also known as propulsion or Saks–Vine [8]. Current studies comparing PULL and PUSH techniques have yielded inconclusive results, with some showing a lower complication rate with the PULL technique [9,10,11], others with the PUSH technique [12], and some finding no statistically significant differences [13,14,15]. Moreover, these studies often focus on comparing the tube insertion technique using a single type of percutaneous approach (endoscopic or radiological) without combining them, despite both approaches appearing equally safe [16].

Placement is not without complications, including bleeding, aspiration, infection, obstruction, perforation, and even death. It is therefore advisable for highly trained teams to carry them out and that gastrostomy is well indicated by specialists in clinical nutrition, avoiding this procedure in patients with a poor prognosis.

The primary objective of this study was to compare complications associated with percutaneous gastrostomies performed using PUSH and PULL techniques, whether endoscopic or radiological, in a tertiary-level hospital.

## 2. Materials and Methods

### 2.1. Study Design

A prospective descriptive observational study was conducted, including all adult patients who underwent percutaneous PULL or PUSH gastrostomy using endoscopic or radiological techniques at the Virgen del Rocío University Hospital in Seville. Subsequently, these patients were followed up in the Nutrition Unit of our center between 2009 and 2020.

The decision as to which technique to use was made on a patient-by-patient basis by the Clinical Nutrition specialist, taking into account that in our center, the endoscopic technique is more widely available for organizational reasons. In the case of a PEG, the patient was admitted to the Digestive System ward. In the case of a PRG, the patient was admitted to the Nutrition ward. It should be noted that in recent years in our center, in patients with amyotrophic lateral sclerosis, radiological gastrostomy procedures have been preferably performed due to the need for less sedation with this technique.

After the gastrostomy was performed, a specialized nurse explained the management of the gastrostomy to the patient and family, and the patient was discharged the following day after checking for acute complications and poor tolerance to enteral formula. Patients and their families were also checked to ensure that they were well instructed before they were allowed to go home.

### 2.2. Gastrostomy Techniques

Endoscopic gastrostomy was performed by highly experienced gastroenterology specialists in endoscopic techniques. In the PULL technique, a thick needle with a mandrel is introduced and its passage into the gastric cavity is observed through the endoscope. The mandrel is removed and a Teflon guidewire is passed through and grasped by a polypectomy loop inserted through the endoscope, and the guidewire attached to the polypectomy loop is removed with the endoscope through the mouth. A dilator attached to the gastrostomy tube is then attached to the Teflon guidewire and PULLed through the mouth, esophagus, and stomach so that it appears by traction through the abdominal wall. At this point, the endoscope is reintroduced at the gastric level to observe that the internal retaining stop of the gastrostomy tube is attached to the gastric wall by placing an external fixation disc or plate. The Sacks–Vine technique (PUSH type) differs in that it uses a Seldinger needle–catheter that penetrates the stomach, through which a metal guidewire is introduced; it is trapped by the polypectomy loop, and the endoscope and the metal guidewire attached to the loop are extracted through the mouth. Then, through the guidewire, the gastrostomy tube is introduced, being attached to a dilator catheter, which will be the first thing to appear through the abdominal wall. From this point on, the techniques are similar. Although our endoscopy team used transiently a Sacks–Vine technique during the first months of endoscopic gastrostomies with a PUSH approach, the vast majority of PUSH-type endoscopic gastrostomies in this study were performed with the Russell technique (also called “introducer PEG”) with three gastropexy points in the following fashion. After identification of a suitable area for gastrostomy placement using transillumination, a chlorhexidine solution is applied to the intact skin. Under aseptic conditions, the area is anesthetized and an 18G needle is introduced into the stomach under endoscopic control. Three gastropexy points are then placed in the area of interest, forming a triangular shape, about two centimeters apart from each other. Although the gastropexy points can be stitched manually, the commercial kits used in our center such as the MIC-KEY^®^ G SAF-T PEXY include semiautomated devices for their implantation. In the virtual center of this triangle, a deep cut about one centimeter long is made with a scalpel. Afterwards, the subcutaneous tissue is dissected with a surgical clamp. Then, an endoscopic guide and dilator with progressive calipers are introduced to mechanically increase the size of the foramen. Later, the inside of the dilator is removed, and the last sheath is kept. After lubricating the tip of the 18 Fr gastrostomy tube, it is introduced inside this sheath, which will be removed hereafter. Finally, the internal balloon of the tube is filled with 10 mL of sterile distilled water, the tube is pulled, and the external stop of the tube is gently lowered until it contacts the skin.

The procedure was carried out in a supine position and had an average duration of 30 min. Due to the need to maintain the supine position and the difficulty in controlling secretions, it was often performed under general anesthesia or monitored anesthetic sedation by an anesthesiology and resuscitation specialist. Additionally, antibiotic prophylaxis was administered. According to protocol, the antibiotic used must be from the cephalosporin group, unless the patient is allergic to this group of drugs. It is administered intravenously half an hour before the procedure. The PULL-type endoscopic technique was employed in our center until 2018, after which the PUSH-type technique began to be used. The latter predominated in 2019 and 2020, although some PULL-type gastrostomies were still performed. 

Radiological gastrostomy was performed by radiodiagnosis specialists highly experienced in interventional radiology, always using the PUSH technique with fluoroscopic guidance and the fixation of the stomach to the abdominal wall with three gastropexy points, in a similar fashion to the Russell technique already described. Prior to this, a computed tomography scan was conducted in all cases to rule out the presence of intestinal loop interposition. Antibiotic prophylaxis was not used in this technique.

The use of surgical gastrostomy in our center is limited (*n* = 29 in a previous analysis performed from 2009 to 2015) [17]. A PULL-type technique associated with endoscopy or “laparoscopic-assisted percutaneous endoscopic gastrostomy” (LAPEG) seems to be the most widespread form of surgical gastrostomy. However, our center has developed a technique that associates surgery with a PUSH-type probe to avoid the use of endoscopy. This technique is called “percutaneous laparoscopic assisted gastrostomy” (PLAG) and has been applied in our center as the main form of surgical gastrostomy since 2010. Because the PLAG technique is not available in all centers, we have not included these surgical gastrostomies in our analysis to improve comparability with previous studies.

### 2.3. Study Variables and Data Collection

The following variables were collected: gender, age, indication for gastrostomy placement (head and neck tumors, esophagogastric tumors, non-tumor esophageal diseases, amyotrophic lateral sclerosis, other neurological diseases, severe malabsorption, maxillofacial diseases, and others), and complications associated with the procedure (deaths due to gastrostomy-associated complications, peritonitis, need for invasive mechanical ventilation, gastrocolic fistula, non-purulent exudate, irritation of the surrounding skin, balloon leakage, obstruction of the gastrostomy tube, stoma dilation, minor bleeding, granuloma, balloon or tube rupture, and/or local infection defined as inflammation and purulent exudate with presence of microorganisms in the culture and need for antibiotic treatment). Data collection, performed by a single researcher, was retrospective, utilizing electronic health records from the Andalusian Health Service through a piece of clinical software called Estación Clínica-Diraya v.4.0.84. Medical records prior to 2010 were collected on paper and subsequently digitized and uploaded to this interactive platform so they could be reviewed on the same workstation. The data were entered into an anonymized Excel database, which was handed over to the principal investigator. This study was approved by our center’s Ethics Committee.

### 2.4. Data Analysis

Statistical analysis was performed using Statistical Package for Social Science (SPSS^®^) version 25 for Windows (IBM Corporation, New York, NY, USA). Descriptive analysis was conducted by obtaining the median and quartiles for quantitative variables (expressed as P50 (P25-P75)) and frequency for qualitative variables [expressed as *n* (%)]. Inferential analysis between the two groups involved Pearson’s chi-square test or Fisher’s test for qualitative variables. Univariate analysis was performed to estimate the association’s magnitude, identifying risk factors for gastrostomy-associated complications.

## 3. Results

A sample of 423 patients was obtained, with 242 undergoing percutaneous PUSH-type gastrostomy (57.21%) and 181 (42.79%) undergoing percutaneous PULL-type gastrostomy. Demographic characteristics are presented in Table 1. Both the percentage of men and the median age were similar between the two groups.

The indications for performing percutaneous PULL and PUSH gastrostomies in our center are displayed in Table 2. For percutaneous PULL gastrostomy, the most frequent indication was neurological diseases (102 (56.4%) patients) followed by head and neck cancer (42 (23.2%) patients). In percutaneous PUSH gastrostomy, the most frequent indication was head and neck cancer (81 (33.5%) patients) followed by amyotrophic lateral sclerosis (68 (28.1%) patients) and neurological diseases (50 (20.7%) patients).

The complications associated with percutaneous PULL and PUSH gastrostomies and their comparison are shown in Table 3. Of note, the percentage of complications was significantly higher in the group of patients who underwent PULL gastrostomy (37.6% vs. 24.8%; *p* = 0.005). The most frequent complication in both groups was exudate, also more frequent in the PULL gastrostomy group than in the PUSH gastrostomy group (18.2% vs. 11.2%; *p* = 0.039). The second most frequent complication was granuloma, although there was no difference between the PULL and PUSH techniques (13.8% vs. 9.5%). Another complication in which there were significant differences, also in favor of a higher frequency in the PULL technique gastrostomy group, was irritation, although this was rare (3.3% vs. 0%; *p* = 0.006). Only one patient died as a result of gastrostomy-associated complications. This occurred in one PUSH gastrostomy patient following an episode of peritonitis. Cases with major complications are described in Table S1.

When univariate analysis was performed to assess risk factors for a higher percentage of complications, no statistical significance was found for gender, age, or any of the possible indications for gastrostomy. Only the indication for ALS had a result close to significance (*p* = 0.092) in favor of an increased risk of complications (Table 4). A multivariate analysis model was used to assess whether age or sex influenced the presence of complications according to techniques, with no significant results. When this same analysis was performed by including each of the gastrostomy indications separately, significant results were obtained when including head and neck tumors (Table 5).

## 4. Discussion

This study evaluates the complication rates associated with all percutaneous PULL and PUSH gastrostomies performed over the last 11 years at a tertiary hospital by highly experienced staff with a nationally recognized Nutrition Unit. The dataset comprises information from 423 patients. To our knowledge, to date, this is the largest observational study with a mixed cohort of both endoscopic and radiologic, PUSH and PULL techniques and diverse clinical indications with the largest number of patients included from the same hospital.

The most common indications for gastrostomy varied depending on the technique employed. For percutaneous PUSH-type gastrostomy, the most frequent causes were head and neck tumors (33.5%), ALS (28.1%), and neurological diseases (20.7%). In contrast, for the PULL technique, neurological diseases were the most common indication (56.4%), followed by head and neck tumors (23.2%), with ALS being less frequent (2.8%). Similar patterns have been reported in other studies regarding the pathologies for which gastrostomies were indicated [18,19]. It is relevant to highlight that when comparing the different techniques performed, it is evident that there is an increase in the indication of these techniques for ALS (since PUSH techniques, which are the most recently implemented, are much more frequent) following the improvement in the care of these patients through multidisciplinary units, with one such unit in our center being a reference at regional level. This upward trend in the indication for PUSH PEG has also been seen in another cohort [20].

Concerning overall complications, our overall complication rate was 30.3%. Among the available endoscopic gastrostomy studies with similar objectives and methodology, the one with the largest sample size is that of Bouchiba et al. [21], with *n* = 854 patients under follow-up after PUSH or PULL PEG. This research found a higher overall complication rate (42.6%), which could be partially explained by the higher prevalence of head and neck cancer in their sample (61.3% vs. 29.1% in our study). As we have already mentioned, the diagnosis of head and neck neoplasms was associated with an increased overall risk of complications with a tendency towards statistical significance, positively attributable to a worse nutritional status at the time of endoscopy compared to other clinical populations with indication for PEG. This would be in line with the study by Retes et al. with *n* = 309 patients with head and neck cancer and PEG implantation (55.7% PULL type, 44.3% PUSH type) and an overall incidence of complications of 36.9%.

Concerning complications separated by PULL vs. PUSH techniques, we found a significantly higher percentage with the PULL technique (37.6% vs. 24.8%; *p* = 0.005). The study by Bouchiba et al. [21] presented a proportion of PEG techniques similar to ours (61.2% PUSH type and 38.8% PULL type vs. 57.2% PUSH type and 42.8% PULL type in our sample), without finding statistically significant differences in the proportion of complications according to PEG type (42.4% with PUSH vs. 42.9% with PULL). Again, these differences could be partially explained by a higher prevalence of head and neck cancer in the PUSH-type PEGs in their sample (89.1% vs. 33.5% in PUSH PEGs in our study and vs. 34.7% in PULL PEGs in their study). These findings differ from those of other retrospective cohort studies—where more complications were observed with the PUSH technique—such as that of Kucka et al. [20] (21.4% vs. 7.1%) in *n* = 1.055 head and neck cancer patients (58.7% PUSH-PEG, 41.4% PULL-PEG), Van Dyck et al. [22] (47.9% vs. 12.1%) in *n* = 57 patients with esophageal or head and neck cancer (42.1% PUSH-PEG, 57.9% PULL-PEG), and Currie et al. [11] (17.1% vs. 7.5%) in *n* = 276 patients with different malignancies (61.2% PUSH-PRG, 37.9% PULL-PRG). Moreover, Currie et al.’s sample included 25.0% proportion of oncological patients with a prior failed endoscopic tube placement attempt, potentially influencing divergent conclusions.

Most complications were minor. A brief comparison with previous studies regarding minor complications is detailed in Table 6. Please note that the definition of minor complications may slightly differ between studies, and that only the most commonly described minor complications have been redacted.

Exudate was the most frequent minor complication in both groups (18.2% in PULL vs. 11.2% in PUSH; *p* = 0.039), followed by granuloma (13.8% vs. 9.5%; *p* = 0.167) and tube leakage (7.2% vs. 5.8%; *p* = 0.561). The PULL technique had a higher rate of local infection (6.6% vs. 3.7%; *p* = 0.173) but a lower rate of bleeding compared to the PUSH technique (0% vs. 0.8%; *p* = 0.327). Although these results did not reach statistical significance, possibly being influenced by the low number of patients experiencing complications, they align with other studies such as that of Retes et al. [23] Additionally, Pih et al. [19] observed a higher number of tube obstructions with the PUSH technique, a difference not observed in our patients.

The PULL technique had a higher rate of local infection (6.6% vs. 3.7%; *p* = 0.173) and granuloma (13.8% vs. 9.5%; *p* = 0.167) but a lower rate of bleeding compared to the PUSH technique (0% vs. 0.8%; *p* = 0.327). Although these results did not reach statistical significance, possibly influenced by the low number of patients experiencing complications, they align with previous studies. Bouchiba et al. [21] found a higher incidence of infected placement sites (8.8% vs. 4.6%; *p* = 0.019) and granulation tissue formation (10.9% vs. 6.9%; *p* = 0.044) in PULL PEG. Unlike us, they found that this technique was also significantly associated with a higher incidence of buried bumper syndrome (7.3% vs. 0.4%; *p* < 0.001). Retes et al. [23] similarly found a higher incidence of local infection (8.7% vs. 4.4%; *p* = 0.131) and granuloma (4% vs. 1.5%; *p* = 0.307) in PULL PEG, which in this case did not reach statistical significance. The PUSH technique occurs under conditions of cutaneous asepsis due to the administration of topical antiseptics. During the PULL technique, a single dose of antibiotic is administered during anesthetic induction to reduce the load of oral, esophageal, and stomach bacterial flora carried by the probe during the procedure. While this antibiotherapy has been shown to reduce the incidence of local infection by reducing the bacterial inoculum [24], complete asepsis is not expected. Therefore, the plausible presence of germs in the tube is the biological substrate that justifies a higher incidence of a local infection that requires antibiotics, or other related signs such as granuloma, exudate, or irritation. It has previously been described that PULL tubes can be more frequently associated with buried bumper syndrome. We believe this is also attributable to the physical design of the catheter, as a plastic disk could exert greater pressure than a distilled water-filled or saline-filled balloon. Nevertheless, we consider that proper handling of the tube is more important to prevent this minor complication. In this regard, we believe that our sample showed no differences because the endoscopy team and clinical nutrition nurses in our center had already completed their learning curve before the study so that they did not over-torque the external stop of the tubes. In addition, proper tube care was ensured by instructing patients and caregivers to rotate the tube daily until it was replaced with a PUSH-type tube.

The PUSH technique had a higher incidence of minor bleeding (0.8% vs. 0%; *p* = 0.327) in our study, both without statistical significance. Again, this trend reached significance in Bouchiba et al.’s paper [21], with a higher incidence of minor bleeding (6.9% vs. 2.1%; *p* = 0.002). Similarly, Retes et al. [23] also found a higher incidence of minor bleeding with PUSH-type PEG (6.6% vs. 0.6%; *p* = 0.006), although these findings are limited because the PUSH group in that study had statistically significantly more advanced baseline disease (98.5% of patients with stage IV head and neck cancer vs. 91.3%). However, the prevalence of anticoagulation or antiplatelet therapy was similar between groups (6.5% vs. 8.1%; *p* = 0.601). Koide’s group [25] also associated PUSH-type PEG as a risk factor for bleeding compared to PULL PEG (OR: 5.236, 95% confidence interval: 1.040–26.316; *p* = 0.045). The use of gastropexy points in the PUSH technique could explain the tendency towards a higher frequency of minor bleeding, These bleedings seem to be associated with puncture of the posterior wall of the gastric greater gastric curvature or with bleeding of the gastroespiploic arteries during fixation of the gastropexy points of PUSH PEGs with the Russell technique or PRGs; however, the group of Suzuki et al. [26] found no differences in bleeding when comparing three and four fixation points (the former being used in our study). The group of Kucha et al. [20] also found a higher incidence of probe displacement in PUSH PEG (14.0% vs. 0.7%). Although we did not find differences in stoma obstruction with the PUSH technique (3.7% vs. 3.9%; *p* = 0.937) and they did not appear in the study by Bouchiba et al. [21] (4.8% vs. 2.7%; *p* = 0.152), other authors have found a higher proportion of probe obstructions with the PUSH technique, such as Retes et al. [23] (10.9% vs. 2.9%, *p* = 0.005) and Pih et al. [19] (14.1% vs. 3.6%; *p* = 0.001).

**Table 6:** Incidence of minor complications (expressed as percentages) in studies analyzing a PUSH vs. PULL approach in non-surgical gastrostomies.

Ref.	This Study	Bouchiba et al. [21]	Retes et al. [23]	Koide et al. [25]	Kucha et al. [20]	Pih et al. [19]	Van Dyck et al. [22]	Currie et al. [11]
**Local infection**								
PULL (%)	6.6	8.8	8.7	11.9	1.7	4.3	9.0	
PUSH (%)	3.7	4.6	4.4	4.1	2.0	4.6	12.0	
*p*-value	0.173	0.019	0.131	0.064	NA	0.903	0.678	
**Granulation tissue formation**								
PULL (%)	13.8	10.9	4.0					
PUSH (%)	9.5	6.9	1.5					
*p*-value	0.167	0.044	0.307					
**Minor bleeding**								
PULL (%)	0	2.1	0.6	1.5	0.2	3.6	0	
PUSH (%)	0.8	6.9	6.6	7.2	1.0	6.9	4.0	
*p*-value	0.327	0.002	0.006	0.037	NA	0.180	0.237	
**Stoma dilation/site leakage**								
PULL (%)	0	5.7	2.9		2.6			1.9
PUSH (%)	1.2	5.2	5.8		1.3			1.2
*p*-value	0.264	0.757	0.257		<0.001			NA
**Buried bumper syndrome**								
PULL (%)	0	7.3	3.5		0.7	0.7		
PUSH (%)	0	0.4	0.7		0	0.4		
*p*-value	NA	<0.001	0.137		NA	1.000		
**Tube obstruction**								
PULL (%)	3.9	2.7	2.9			3.6		1.9
PUSH (%)	3.7	4.8	10.9			14.1		1.2
*p*-value	0.937	0.152	0.005			0.001		NA

NA = Not applicable.

Regarding major complications, there were five cases of peritonitis, with more cases associated with the PULL technique, though not reaching statistical significance (2.8% vs. 0.8%; *p* = 0.187). These findings are consistent with frequencies reported in other studies. Although there were no differences between groups in terms of peritonitis in the study of Bouchiba et al. [21], PULL PEG had a higher incidence of perforation with a trend toward statistical significance (0.9% vs. 0%, *p* = 0.058). These findings are consistent with frequencies reported in other studies. Additionally, three gastrocolic fistulas occurred, one with the PULL technique and two with the PUSH technique (*p* = 0.608), a rare complication with few reported cases in the literature [27,28,29].

It is worth noting that despite the large number of patients who underwent gastrostomy, we report a solitary death case. This involved a patient with a well-differentiated pharyngeal carcinoma, causing upper airway obstruction with a substantial tumor mass, who presented with peritonitis following a PRG with gastropexy using the PUSH technique. This datum contrasts with findings from other large cohort studies, such as that of Pih et al. [19], comprising a sample size of 401 patients, who recorded 20 deaths within 30 days of undergoing percutaneous endoscopic gastrostomy between 2005 and 2015. Earlier studies, like that of Platt et al. [28], reported lower figures (3 deaths), which are more in line with our data. In the study of Bouchiba et al. [21], two deaths occurred exclusively in PULL PEG, whereas in that of Kucha et al. [20], one occurred in PUSH PEG and one in PULL PEG.

In our study, both ALS and gastroesophageal tumors showed results close to significance when studying their behavior as risk factors for gastrostomy-associated complications. In fact, a prior study with a 75.9% prevalence of head and neck cancer found that PEG placement during active chemoradiotherapy treatment was a risk factor for major adverse events (HR = 2.73; 95% CI: 1.03–7.20; *p* = 0.042) in comparison with PEG placement after treatment [20], possibly due to worsened nutritional status. This aligns with findings by Lee et al. [30], who also found no clear association between these gastrostomy indications and the risk of complications, although they associated neurological diseases with a higher complication rate, which was inconsistent with our results. Other studies, such as that of Park et al. [16], also observed more complications in patients with neurological diseases.

The main limitation of our study is its observational and retrospective nature. All PULL and PUSH gastrostomies were included, regardless of whether they were performed endoscopically or radiologically, which could introduce bias. However, as mentioned earlier, published data to date indicate the similar safety of both techniques. Additionally, performing a study with such a long follow-up period may introduce historical bias, as the endoscopic PUSH technique was implemented in our center in 2018, although the radiological PUSH technique coincided in time with the PULL technique.

Nevertheless, some strengths should be highlighted, such as the large number of gastrostomies performed in a single center, carried out by experienced endoscopists and interventional radiologists, and the subsequent patient follow-up in a Nutrition Unit with a high volume of gastrostomy patients maintaining consistent protocols over the years; these factors increased the internal consistency of our results.

We consider the publication of our results to be relevant further evidence on the safety of gastrostomy procedures in general. Furthermore, we believe that the complications associated with the different gastrostomy techniques should be published in order to implement improvements, as this is a field in continuous development. Finally, our experience as a reference center may be useful to other colleagues when deciding which type of gastrostomy to perform in different patients, and it will also be useful on the clinical management level to make decisions on the possible implementation of new techniques in service portfolios depending on the hospital or center.

## 5. Conclusions

Percutaneous PULL gastrostomies were associated with a higher percentage of complications than PUSH gastrostomies at our center between 2009 and 2020. Nevertheless, based on our results, we conclude that both techniques are safe, with the majority of complications being minor. Among these, exudate, granuloma, and tube leakage were the most frequent. Both exudate and irritation were more common with the PULL technique. The most frequently indicated pathologies were neurological diseases, head and neck cancer, and ALS. Conducting and publishing studies on safety and comparison between different gastrostomy techniques is crucial for their improvement and the subsequent reduction of complications.

## Figures and Tables

**Table 1:** Demographic characteristics of patients undergoing percutaneous PULL-type vs. PUSH-type gastrostomy at Virgen del Rocío University Hospital between 2009 and 2020.

	Percutaneous PULL Gastrostomy (*n* = 181)	Percutaneous PUSH Gastrostomy (*n* = 242)
Male gender	332 (64.8%)	158 (65.3%)
Age (years)	61 (41–71)	63 (56–72.7)

**Table 2:** Indication for percutaneous PULL or PUSH gastrostomy using endoscopic or radiological techniques at Virgen del Rocio University Hospital between 2009 and 2020.

Indication	Percutaneous PULL Gastrostomy (*n* = 181)	Percutaneous PUSH Gastrostomy (*n* = 242)
Head and neck cancer	42 (23.2%)	81 (33.5%)
Esophagogastric cancer	5 (2.8%)	20 (11.6%)
Non-tumor esophagogastric pathology	3 (1.7%)	6 (2.5%)
ALS (amyotrophic lateral sclerosis)	5 (2.8%)	68 (28.1%)
Neurological diseases	102 (56.4%)	50 (20.7%)
Severe malabsorption	3 (1.7%)	1 (0.4%)
Maxillofacial pathology	1 (0.6%)	1 (0.4%)
Other indications	18 (9.9%)	10 (4.1%)

**Table 3:** Complications associated with percutaneous PULL vs. PUSH gastrostomy using endoscopic or radiological techniques at Virgen del Rocío University Hospital between 2009 and 2020.

Complication	Percutaneous PULL Gastrostomy (*n* = 181)	Percutaneous PUSH Gastrostomy (*n* = 242)	*p*-Value
Complications	68 (37.6%)	60 (24.8%)	0.005 ^a^
Deaths due to gastrostomy-related complications	0 (0%)	1 (0.4%)	0.572 ^b^
Peritonitis	3 (2.8%)	2 (0.8%)	0.187 ^b^
Need for invasive mechanical ventilation after the procedure	0 (0%)	0 (0%)	N.A.
Gastrocolic fistula	1 (0.5%)	2 (0.8%)	0.608 ^b^
Exudate	33 (18.2%)	27 (11.2%)	0.039 ^a^
Irritation	6 (3.3%)	0 (0%)	0.006 ^b^
Tube leakage	13 (7.2%)	14 (5.8%)	0.561 ^a^
Obstruction	7 (3.9%)	9 (3.7%)	0.937 ^a^
Stoma dilation	0 (0%)	3 (1.2%)	0.264 ^b^
Bleeding	0 (0%)	2 (0.8%)	0.327 ^b^
Granuloma	25 (13.8%)	23 (9.5%)	0.167 ^a^
Rupture	8 (4.4%)	5 (2.1%)	0.165 ^a^
Local infection	12 (6.6%)	9 (3.7%)	0.173 ^a^
Buried bumper syndrome	0%	0%	N.A.

^a^ X2 test; ^b^ Fisher’s test; N.A. = not applicable.

**Table 4:** Risk factors for total complications associated with percutaneous PULL and PUSH gastrostomies using endoscopic or radiological techniques performed at Virgen del Rocio University Hospital between 2009 and 2020.

	Univariate Analysis		
	OR	95% CI	*p*-Value
**Female gender**	0.847	(0.553–1.297)	0.444
**Age**	1	(0.973–1.027)	0.98
**Head and neck cancer**	1.508	(0.966–2.355)	0.071
**Esophagogastric cancer**	0.741	(0.353–1.556)	0.428
**Non-tumor esophagogastric pathology**	0.534	(0.141–2.024)	0.356
**ALS**	1.676	(0.922–3.049)	0.091
**Neurological diseases**	1.346	(0.866–2.094)	0.187
**Severe malabsorption**	0.142	(0.015–1.376)	0.092
**Maxillofacial pathology**	0.432	(0.027–6.96)	0.554
**Other indications**	0.91	(0.4–2.07)	0.882

**Table 5:** Multivariate analysis including percutaneous gastrostomy technique, sex, and age.

	Univariate Analysis		
	OR	95% CI	*p*-Value
**Female gender**	0.318	(0.113–0.894)	0.003
**Age**	0.993	(0.965–1.023)	0.662
**PUSH gastrostomy**	0.898	(0.33–2.444)	0.833
**Head and neck cancer**	1.081	(0.946–1.112)	0.948

## Data Availability

The data presented in this study are available on request from the corresponding author.

## Supplementary Materials

The following supporting information can be downloaded at: https://www.mdpi.com/article/10.3390/jcm13071836/s1, Table S1: Description of cases with major complications.
