# Supplementary material for: Percutaneous Gastrostomies: Associated Complications in PUSH vs. PULL Techniques over 12 Years in a Referral Centre
Source: J Clin Med. 2024 Mar 22;13(7):1836. doi: 10.3390/jcm13071836 (PMC11012573; doi:10.3390/jcm13071836)
Supplement: Supplementary file 1 [file jcm-13-01836-s001.zip › jcm-2885908-supplementary.pdf]

Supplemental Table S1. Description of cases with major complications

Peritonitis case # 1<br>Male, 52 years old<br>Underlying disease: Tonsil carcinoma cT4a cN2b cMx (stage IVA), naïve<br>Gastrostomy technique: PEG PULL<br>Description: diagnosed with acute abdomen the day after PEG. An urgent abdominal computed tomography (CT) scan was performed, showing a large pneumoperitoneum. He underwent emergency surgery that day. A midline laparotomy was performed, with cleansing of the abdominal cavity and conversion to a Stamm gastrostomy. As an immediate complication of the surgery, he presented a surgical wound infection, initially treated intravenously in the hospital and finally via PEG at home. Subsequently, he presented dehiscence of the laparotomic suture and evisceration one month later, being reintervened with an abdominal wall suture and mesh reinforcement. No further complications.
Peritonitis case # 2<br>Female, 31 years old<br>Underlying disease: neonatal encephalopathy with Lennox-Gastaut syndrome.<br>Gastrostomy technique: PEG PULL<br>Description: diagnosed with acute abdomen three days after PEG. An urgent abdominal CT scan was performed, showing a large pneumoperitoneum and an intra-abdominal abscess. She underwent emergency surgery that day by midline laparotomy with cleansing of the abdominal cavity, abscess drainage with sampling for microbiology and conversion to a Janeway gastrostomy. No further complications.
Peritonitis case # 3<br>Female, 42 years old<br>Underlying disease: post-anoxic encephalopathy after cardiorespiratory arrest.<br>Gastrostomy technique: PEG PULL<br>Description: diagnosed with acute abdomen the day after PEG. An urgent abdominal CT scan was performed, showing pneumoperitoneum. She underwent emergency surgery that day by midline laparotomy with cleansing of the abdominal cavity and conversion to a Stamm gastrostomy. The surgical procedure revealed a tumor involving the cecal appendix, cecum, and ileocecal fold, with multiple lymphadenopaties throughout. Therefore, a right hemicolectomy was added with section of the transverse colon and ileum with latero-lateral isoperistaltic anastomosis. The final histological diagnosis was a poorly differentiated adenocarcinoma of the cecum G3 pT4b N2b Mx. Next year, the patient developed a left colon adenocarcinoma pT2N1Mx intervened by left hemicolectomy. A genetic test was performed, with a final diagnosis of Lynch syndrome.
Peritonitis case # 4<br>Male, 59 years old<br>Underlying disease: oropharyngeal carcinoma T2 N3 M0, treated with chemotherapy and radiotherapy.<br>Gastrostomy technique: PRG PUSH.<br>Description: the immediate procedure was uneventful. On arrival at the ward, it was found that the internal fixation balloon was broken, although gastropexy points seemed untouched. On the third day, he was diagnosed with acute abdomen. Systemic antibiotherapy was started and a new urgent PRG was performed using the existent stoma. With a favorable evolution and no further incidents, he was discharged with outpatient antibiotherapy, which he completed via PRG.
Peritonitis case # 5<br>Male, 61 years old<br>Underlying disease: well-differentiated pharyngeal carcinoma cT4N3Mx.<br>Gastrostomy technique: PRG PUSH.<br>Description: the initial procedure was uneventful. He was diagnosed with an acute abdomen the day after PRG. An urgent abdominal CT scan was performed, showing dehiscence of gastropexy points and pneumoperitoneum. He later developed an upper airway obstruction with exitus the same day.
Gastrocolic fistula case # 1

Male, 44 years old<br><br>Underlying disease: Huntington's disease, with severe cognitive impairment and neurogenic dysphagia.<br><br>Gastrostomy technique: PEG PULL<br><br>Description: it was impossible to obtain a point of contact between stomach and abdominal wall by transillumination and digitopressure with endoscopy, so a direct visualization of the abdominal cavity was added with a laparoscopic camera. One year later, it was impossible to replace the gastrostomy tube. An abdominal CT scan was performed, showing transverse colon interposed between the stomach and the abdominal wall, with a gastrocolic fistula. Via laparoscopic surgery the gastrostomy tube was removed, colon and stomach orifices were closed, and a Janeway gastrostomy was performed, with no further complications.
Gastrocolic fistula case # 2<br><br>Female, 58 years old<br><br>Base disease: Niemann-Pick disease with neurogenic dysphagia.<br><br>Gastrostomy technique: PEG PULL<br><br>Description: the initial procedure was uneventful. One year later, fecal contents were observed coming out through the gastrostomy orifice during the endoscopic replacement of the tube. An abdominal CT scan was performed, which revealed severe dilatation of the colic framework up to the rectosigma with abundant fecaloid debris, without images of pneumoperitoneum. Via laparoscopic surgery the gastrostomy tube was removed, colon and stomach orifices were closed, and a Janeway gastrostomy was performed, with no further complications.
Gastrocolic fistula case # 3<br><br>Male, 73 years old<br><br>Underlying disease: infiltrating epidermoid carcinoma of the oropharynx G1 cT4b cN2 treated with chemotherapy and radiotherapy.<br><br>Gastrostomy technique: PRG PUSH<br><br>Description: during oral endoscopy, bleeding occurred when dilating a stricture in the upper esophageal portion, so the procedure was abandoned and PRG was performed without immediate incidents. Ten months later, after replacement of the gastrostomy tube, she presented with diffuse colicky abdominal pain and episodes of diarrhoea. An urgent abdominal CT scan located the distal end of the gastrostomy tube inside the lumen of the distal segment of the transverse colon, with discrete distension and pneumatization of the latter. Via laparoscopic surgery the gastrostomy tube was removed, colon and stomach orifices were closed, and a Janeway gastrostomy was performed, with no further complications.
